# Perceptions and Experiences of Heart Failure Patients and Clinicians on the Use of Mobile Phone-Based Telemonitoring

**DOI:** 10.2196/jmir.1912

**Published:** 2012-02-10

**Authors:** Emily Seto, Kevin J Leonard, Joseph A Cafazzo, Jan Barnsley, Caterina Masino, Heather J Ross

**Affiliations:** ^1^Centre for Global eHealth InnovationUniversity Health NetworkToronto, ONCanada; ^2^Department of Health Policy, Management and EvaluationUniversity of TorontoToronto, ONCanada; ^3^Institute of Biomaterials and Biomedical EngineeringUniversity of TorontoToronto, ONCanada; ^4^Department of MedicineUniversity of TorontoToronto, ONCanada; ^5^Divisions of Cardiology and TransplantUniversity Health NetworkToronto, ONCanada

**Keywords:** heart failure, telemonitoring, mobile phone, patient monitoring, self-care, qualitative research

## Abstract

**Background:**

Previous trials of heart failure telemonitoring systems have produced inconsistent findings, largely due to diverse interventions and study designs.

**Objectives:**

The objectives of this study are (1) to provide in-depth insight into the effects of telemonitoring on self-care and clinical management, and (2) to determine the features that enable successful heart failure telemonitoring.

**Methods:**

Semi-structured interviews were conducted with 22 heart failure patients attending a heart function clinic who had used a mobile phone-based telemonitoring system for 6 months. The telemonitoring system required the patients to take daily weight and blood pressure readings, weekly single-lead ECGs, and to answer daily symptom questions on a mobile phone. Instructions were sent to the patient’s mobile phone based on their physiological values. Alerts were also sent to a cardiologist’s mobile phone, as required. All clinicians involved in the study were also interviewed post-trial (N = 5). The interviews were recorded, transcribed, and then analyzed using a conventional content analysis approach.

**Results:**

The telemonitoring system improved patient self-care by instructing the patients in real-time how to appropriately modify their lifestyle behaviors. Patients felt more aware of their heart failure condition, less anxiety, and more empowered. Many were willing to partially fund the use of the system. The clinicians were able to manage their patients’ heart failure conditions more effectively, because they had physiological data reported to them frequently to help in their decision-making (eg, for medication titration) and were alerted at the earliest sign of decompensation. Essential characteristics of the telemonitoring system that contributed to improved heart failure management included immediate self-care and clinical feedback (ie, teachable moments), how the system was easy and quick to use, and how the patients and clinicians perceived tangible benefits from telemonitoring. Some clinical concerns included ongoing costs of the telemonitoring system and increased clinical workload. A few patients did not want to be watched long-term while some were concerned they might become dependent on the system.

**Conclusions:**

The success of a telemonitoring system is highly dependent on its features and design. The essential system characteristics identified in this study should be considered when developing telemonitoring solutions.

**Key Words:**

## Introduction

Telemonitoring systems have been developed with the goal to improve outcomes and reduce the high costs associated with heart failure. However, the results from previous trials have been inconsistent, largely due to the diverse interventions under investigation and varying study designs [1-6]. There is currently a lack of insight into the features required for successful telemonitoring solutions.

A mobile phone-based telemonitoring system was developed with a user-centered design process, including iterative rounds of usability testing with heart failure patients and clinicians. The telemonitoring system was then evaluated through a randomized controlled trial (ClinicalTrials.gov NCT00778986) with 100 heart failure patients (n = 50 in each of the intervention and control groups). The primary intent of the trial was to pilot the telemonitoring system in order to determine the impact of the system on self-care, clinical management, and health outcomes. The quantitative findings from the trial suggested that the telemonitoring system improved quality of life through improved self-care and clinical management. The quantitative results are presented in detail in an accompanying paper published in this issue [7].

This paper discusses the qualitative findings from the trial based on in-depth patient and clinician interviews. The objective of the qualitative analysis was to obtain an understanding of the effects of the mobile phone-based telemonitoring system on self-care and clinical management. In addition, the analysis provides insight into the characteristics of a successful telemonitoring solution.

## Methods

### Study Design

The heart failure patient participants were recruited from the University Health Network (UHN) Heart Function Clinic in Toronto, Ontario, between September 2009 and February 2010. Semi-structured interviews were conducted with 22 heart failure patients who had used the telemonitoring system for 6 months. To be eligible for participation in this study, patients had to be older than 18 years of age, not be on the heart transplantation list, be expected to survive more than one year, and have a left ventricular ejection fraction (LVEF) < 40%. The UHN Research Ethics Board approved the study prior to commencement.

Although 50 heart failure patients used the telemonitoring system in total, saturation from the interview data was achieved after 22 patients. The choice of interviewees was generally based on who was first scheduled to visit the Heart Function Clinic, because the interviews were conducted face-to-face at their first scheduled clinic visit 6 months after recruitment. Efforts were made to interview patients who have had differing experiences with the telemonitoring system.

The five clinicians (three cardiologists and two nurse practitioners) from the Heart Function Clinic who interacted with the telemonitoring system during the study were also interviewed post-trial. Each patient and clinician semi-structured interview was between 15 and 60 minutes in duration. All interviews were audio recorded and later transcribed.

### Study Intervention

The participants were provided with the telemonitoring system, in addition to standard care. They were asked to use the telemonitoring system to take daily morning weight and blood pressure readings, and to answer daily morning symptom questions (mainly yes/no questions) on a mobile phone for 6 months. The 17 patients who did not have an implantable cardioverter-defibrillator (ICD) were provided with an ECG recorder and asked to take weekly recordings (ECG recorder was not certified for use with ICDs). The weight and blood pressure readings (UA UC-321PBT weight scale and UA-767PBT blood pressure monitor, A&D Medical, USA) and ECG recordings (SelfCheck ECG PMP4, CardGuard, Israel) were automatically sent wirelessly via Bluetooth to a mobile phone (BlackBerry Pearl 8130, Research in Motion, Canada). A custom-designed and -built software application on the mobile phone was used to display and store the data, and to transmit the information to the data repository at the hospital.

Standard care at the UHN Heart Function Clinic included visits to the clinic from once every 2 weeks to once every 3-6 months, depending on the severity of the patient’s heart failure condition and the need for optimization of their medication. Standard care also included heart failure education during preliminary visits at the Heart Function Clinic and the ability to telephone the clinic as necessary.

An instruction or alert was sent to the mobile phone based on all the physiological and symptom information. Both the patients and clinicians were able to view the data on a secure website. All data were also stored and accessible on the mobile phone. If a patient did not take all the required measurements by 10 AM each day, an automated adherence reminder phone call was sent to their home telephone. The use of the home telephone number as a contact point was deemed preferable, in case the patient was not near the system, the BlackBerry was in another room, the BlackBerry was turned off, etc. Patients in the telemonitoring group were given an individual training session on how to use the system and were provided with the telemonitoring equipment to take home during the recruitment session. Participants could also access technical support by phone throughout the study. Alerts with all the relevant information, including the ECG recording as an attachment, if available, were emailed to the physician’s mobile phone, if measurements were outside the target range or symptoms were reported.

### Data Analysis

The transcripts were analyzed using a conventional content analysis approach [8]. The study coordinator and a second reviewer independently analyzed and coded the transcripts with the software program NVivo version 7 (QSR International, Doncaster, Victoria, Australia). Key themes were identified by both researchers and then discussed until agreement on the themes was reached. For the patient post-trial interview data, member checking was performed with six telemonitoring group participants who had agreed to participate in the post-trial interviews. Efforts were made to interview patients who had differing experiences with the telemonitoring system. The six participants were mailed a summary of the themes from the qualitative analysis and then called at home a week later to discuss their thoughts on the themes.

## Results

The demographic and clinical characteristics of the 22 interviewed participants were representative of the patient population attending the UHN Heart Function Clinic, including an average age of 57 (SD 14) and 82% (n = 18) male. The following is a discussion of the themes found from the conventional content analysis.

### Increased Self-Care

The telemonitoring system enabled patients to appropriately modify their lifestyle behaviors (eg, salt and fluid restrictions, diuretic dose, and exercise) at the first sign of decompensation. Both the automated instructions from the telemonitoring system and the clinician phone calls during times of apparent decompensation provided timely feedback and reinforcement on what was the most appropriate course of action, including taking extra diuretic medication. Thus, patients received the instructions during “teachable moments”. A “teachable moment” is when the ability to learn a particular task is possible, because the timing is right [9]. These improvements in self-care were enabled through several factors related to the use of the telemonitoring system as described below.

#### Improved Awareness and Knowledge of Heart Failure Condition

Patients expressed becoming more aware of their heart failure condition and their own body, because they were taking their physiological measurements and symptoms daily, and their weight and blood pressure targets were brought to their attention daily. The automated feedback and clinician phone calls also alerted the patients when their health appeared to be worsening.

Patient #16 …I never thought of my sodium. (The telemonitoring system) made me look at other things (even though) I thought I was healthy. (The cardiologist) made me more aware of my sodium levels by my weight. My weight was fluctuating and she said, ‘You're using too much salt. Your blood pressure is a little high, you know.’ I'm even more aware now than I was before I started this program.

The patients were also more aware of the cause and effect relationship between their lifestyle choices and their health. In particular, they were able to correlate diet, fluid consumption, medication adherence, and exercise with changes in their weight, blood pressure, and symptoms.

Patient #2 …It's really taught me what the correlation is between salt intake and weight and water retention. An above normal sodium intake will show up immediately the next day as a weight gain and then as you clear that out of your system it goes back.

#### Increased Reassurance/Reduced Anxiety

Patients expressed feeling reassured that someone was watching over them. Some patients had substantial anxiety prior to using the telemonitoring system, especially those who were newly diagnosed or those who recently had an acute cardiac episode. Many patients referred to the telemonitoring system as a “security blanket” and it was “like almost having a doctor right beside you”. Patients stated that after taking their daily measurements in the morning, they could go about their day without worry, if the telemonitoring system sent them a message confirming that everything was normal. The patients’ informal caregivers (ie, family members) also felt reassured by the telemonitoring system.

Patient #33 …I was in the hospital five times last year, because of anxiety and my potassium getting out (of control). That did not happen since the study. So, it saves trips to the clinic for sure and it removes anxiety.

The patients also felt more reassured, because they were more connected to their healthcare team and their clinicians had more information about their condition. They believed they would not “fall through the cracks” as they did before using the telemonitoring system.

Patient #2 …It tends to eliminate one of the biggest problems of being sick and that's a sense of isolation, because I know that there's regular (ongoing) contact. So, if I'm not feeling well, I know I’m going to be getting a phone call and it seems to me that's worth gold.

#### Increased Empowerment and Confidence

Patients expressed feeling more in control, confident, and accountable, because they could directly observe the effects of their lifestyle choices on their health and become active participants in their own health. As noted above, many patients learned the correlation between consuming sodium and changes in weight and blood pressure. Some patients also received automated reminders to take extra diuretic medication after a weight gain, which confirmed taking the extra medication was the correct course of action. This group of patients received prior instruction from their cardiologist to take extra diuretic medication in this situation, but they still often felt uncertain of making the decision to take the extra medication on their own.

Patient #9 …I think I'm more responsible for myself, or more accountable I should say, because I know that I have to send this in and then I look at it. If my weight is higher, then I make sure that I make changes in the diet or make changes in the food restrictions that I'm supposed to and, if it's low, then I can also make (the) changes that I need.

#### Increased Self-Care Motivation

Patients expressed being more motivated to adhere to the recommended daily monitoring of weight, blood pressure, and symptoms owing to the telemonitoring system for several reasons. First, the patients knew their clinicians would find out, if they did not perform their daily measurements. This provided an incentive for the patients to perform the measurements, because they did not want their healthcare providers to think they were not following their instructions. Second, the patients expressed sensing that their measurements were now being used and interpreted. Prior to the trial, some of the patients stopped taking and recording their measurements, especially blood pressure readings, because they did not know how to interpret the values themselves and clinicians were not reviewing them.

Patient #13 …The fact that I get a call (about) my weight, that means somebody is really looking at this, so that was a bit of a confidence booster. The fact that this isn't just being chucked.

Third, the telemonitoring system helped them to establish a habit of taking measurements first thing in the morning.

Patient #18 …I kind of liked the fact that I was sort of following a routine and actually checking my weight and my blood pressure. It sort of gave me some comfort that I was sort of doing that kind of stuff. It's all those doubts that go through your mind. Having some routine sort of stabilizes myself in the morning.

### Improved Clinical Management

The clinicians believed telemonitoring improved clinical management, because the system provided increased patient data for decision support and the automated alerts notified them at the earliest sign of decompensation.

#### Patient Data for Clinical Decision Support

The clinicians thought the telemonitoring system provided comprehensive information to support clinical decision-making. For example, the real-time daily weight, blood pressure, and symptom data were used to initiate 105 additional medication adjustments and instructions over the six-month trial. Of particular importance was a statistically significant increase in the number of patients in the telemonitoring group who were prescribed an aldosterone antagonist (type of diuretic medication) during the trial.

Previous studies have found less than a third of eligible patients receive heart failure guideline-recommended aldosterone antagonist therapy, partially because of the need to closely monitor serum potassium levels, due to the risk of hyperkalemia (a potentially fatal condition from elevated concentration of potassium in the blood) [10, 11]. The increased use of aldosterone antagonists in the telemonitoring group may reflect the ability of clinicians to closely follow their patients' daily weights and blood pressures, enhancing the feeling of security with medication up-titration. Patients could be easily instructed to see their primary care physician for follow-up of serum potassium levels as necessary. The involvement of the primary care physician was not excluded in the trial. All patients seen in the Heart Function Clinic have routine follow-up with their primary care physician or community cardiologist. Communication is maintained after all clinic visits and telephone communication is encouraged for issues or clinical concerns. The system could be easily adapted to allow monitoring by a nurse practitioner, primary care physician, internist, or community cardiologist.

Cardiologist #2 …I think it was a very useful tool and that it has complemented the usual care that we provide to our patients. It was a way to detect things that we don't usually or we are unable to detect, because of ambulatory issues that some patients do not report. In many cases, it helped us make decisions, admissions, change of medications, closer follow up, and we have changed, a little bit, the (care) plan.

#### Alerts of Patient Decompensation

The clinicians believed the telemonitoring system enabled them to detect decompensation earlier, because of the alerts generated from the daily patient physiological and symptom data. The clinicians considered the ability to individualize the system for different patients to be very important (ie, adjusting their target values). They also thought the system prompted them to interact with their patients at the most appropriate time in order to reinforce appropriate behaviors (eg, reduction of salt intake or extra diuretic medication after a sudden weight gain).

Cardiologist #2 ...Absolutely, it's a learning tool. At the beginning of this, they had no idea that their weight can change and they have a target. They learn, because they received the call: ‘okay, you gained three pounds, take an extra lasix’ [diuretic medication]. So, they know that, and in the future they will do it by themselves.

### Other Perceived Benefits

Both patients and clinicians were motivated to continue using the telemonitoring system, because of the perceived benefits. Besides the improved self-care and clinical management discussed above, there was a perception telemonitoring would reduce clinic visits and hospitalizations. The ease of use and portability of the system were also found to be benefits of the mobile phone-based telemonitoring system.

#### Reduction in Clinic Visits and Hospitalizations

Reducing the number of trips to the Heart Function Clinic was one of the most commonly cited potential benefits by patients. This was especially important for patients who lived in areas far from the clinic, felt very ill, and found coming to the clinic to be a financial burden. Although the trial was underpowered to detect changes in number of hospitalizations, clinicians believed telemonitoring could reduce the rate of hospitalizations, which could lead to cost savings. They mentioned that preventing one hospital admission per patient could pay for the cost of the telemonitoring system.

Cardiologist #3 …The cost effectiveness (determined from) the number of hours and effort, and the cost of retaining the system (versus the savings from reduced hospitalization) is to be determined, but my sense is it definitely will save the department and the hospital (money).

#### Ease of Use

The telemonitoring system required the patients to take only a few minutes each morning to send in the physiological values. None of the participants had expressed issues using the system. Special consideration for those with vision impairment was accommodated in the design of the application, such that information was displayed with a large typeface and high contrast (black lettering on a white background, with no extraneous information). Even very elderly patients (oldest study participant was 88 years old) and those with no mobile phone experience were able to successfully use the telemonitoring system. Some patients required up to a week to become comfortable using the system or phoned for technical support, especially those who were less technologically savvy or who were not completely fluent in English. Many patients also had family members who could help them when necessary.

Patient #33 …That's a big advantage, because now they're not having to learn how to use a modem and anything else. (The mobile phone) gets a reading, if it seems like a valid reading, off it goes.

#### Portability

Patients commented that the portability of the mobile phone-based telemonitoring system was useful, because it could be taken on vacation or to the cottage. The available website was seldom used by patients (only 13 patients ever logged onto the website), because all the relevant information was displayed on the mobile phone. Therefore, patient access to a computer was unnecessary.

Patient #18 ...It gave me a lot of peace of mind, particularly (when) I was in Florida without a doctor nearby for three months, and it was just wonderful to know that my vital signs were being monitored. I'm very, very impressed at the responses that I got for those few times that the results were not within the guidelines.

Clinicians liked the portability of the telemonitoring system, because they could monitor their patients from anywhere since the alerts were sent to their mobile phones. The website was also infrequently used by the clinicians, because the email alerts usually provided all the required information and enabled the clinician to simply click on the displayed patient’s phone number to dial the number.

Cardiologist #1 …I was at my cottage or when I was away on a weekend, I was always able to be (informed about my patients). I was in Bermuda and calling patients from Bermuda when I was down there on a four-day holiday weekend, and I would get a hold of the patients and stay on top of things.

### Barriers to Long-Term Implementation

The large majority of patients wanted to continue to use the telemonitoring system indefinitely. The clinicians wanted to integrate the system into the Heart Function Clinic. However, there were several concerns raised regarding its long-term use.

#### Ongoing Costs

The clinicians and patients indicated cost was one of the main barriers to implementing the telemonitoring system on a long-term basis, although the cost to deliver this mobile phone service is projected to be much lower than conventional telemonitoring systems. In general, patients thought the healthcare system should pay for the use of the telemonitoring system through the Ontario Health Insurance Plan (OHIP), because it would reduce healthcare system costs in the long run. Patients were asked if they would be willing to pay for the telemonitoring system (ie, equipment and monthly cellular phone/data charges). Some patients were willing to partially pay for the use of the telemonitoring system, if it was a reasonable amount. In fact, some patients offered to pay at the end of the trial to be able to keep using the telemonitoring system.

Patient #33 …I think the healthcare system is going to save money. I don't know what the costs are of those devices, but I know that I have not been in the emergency department over night or for a period of time since we started this. Of course, everybody would like OHIP to pay for it. In my case, it’s worth money to me and I would (pay for it). You’d be afraid not to do it.

Some patients suggested having to pay a nominal fee might even encourage patients to adhere to taking the measurements.

Patient #18 ...If I had to pay five dollars or ten dollars every month to be on this system, then I think it would keep me more consistent and saying, ‘okay, I'm paying for this thing, I might as well do it’. They go, ‘if it's not costing me anything, I don't care’, whereas if it costs you something you tend to pay a little more attention to it.

However, some patients indicated they did not have the financial means to help pay for using the telemonitoring system. Patients also suggested insurance companies or work benefits might be able to help fund the telemonitoring system. A few patients mentioned patients might be able to claim the use of the telemonitoring system as a medical expense on their income taxes, but it would be only a small return.

Patient #34 …Willing to pay is a hard question, as some people are on disability and cannot afford it.

**Figure 1:**
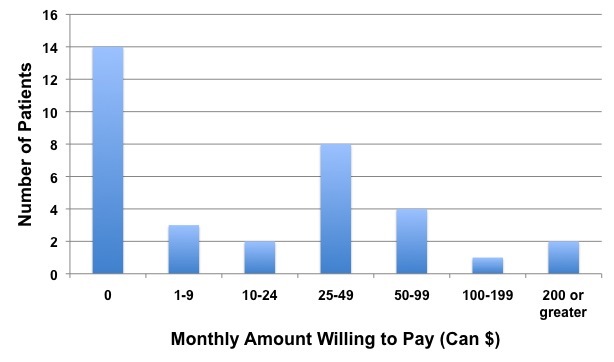
Responses to amount per month patients would be willing to pay to use the telemonitoring system.

Participants were asked how much they would be willing to pay per month to continue using the telemonitoring system. Figure 1 displays the responses to this question. Fourteen patients responded they would not pay to use the telemonitoring system, but many of these patients stated in the comment section that they did not have the financial means to pay and the healthcare system should fund it. The second most common response was paying between Can $25 and Can $49 per month (8 respondents).

#### Increased Clinical Work Load and Workflow Changes

One of the cardiologists’ main concerns was the potential lack of time to respond to alerts, due to their busy schedules, if the telemonitoring system was permanently integrated into the clinic. They suggested a nurse practitioner manage the alerts, instead of a cardiologist.

Cardiologist #3 …I think it's ideally suited for a nurse practitioner. Most of the issues you're dealing with are straightforward. They really probe into what a patient has been up to, and they kind of scratch below the surface more.

The nurse practitioners were in favor of managing the alerts themselves. However, it was recognized that a nurse practitioner would need dedicated time to respond to the alerts and should become familiar with the patients on the telemonitoring system. The vast majority of the alerts would be generated in the morning after patients take their daily measurements. For nights and weekends, the cardiologist on call could respond to the alerts. The email alerts could be sent to a specific mobile phone that could be passed between the clinicians managing the alerts.

Nurse Practitioner #1 …I'd have no problems (managing the alerts). I think that would be great. I now communicate very closely with patients around their weight and lasix dosing and stuff like that. So, they call me directly and sometimes I'll talk to them daily. I find that as a result of that, they feel better connected.

#### Readiness of the Clinic for Integration

A challenge to integrating the telemonitoring system into the clinic would be maintaining up-to-date blood test values and medication, in order to provide current information in the email alerts to the clinicians. The hospital’s electronic health record (EHR) was not always up-to-date (eg, outside blood test results and changes in medication between clinic visits were recorded on a paper chart). In addition, the data were usually in a free text clinical note that could not be easily used to populate a database. Therefore, changes in medication and blood test values were manually entered into a separate telemonitoring system database for the trial and manually updated monthly by a clinical research fellow. Ideally, the telemonitoring system and EHR should be integrated in the future and all relevant information should be available electronically.

Cardiologist #3 …If someone had blood work done a week ago or even two weeks ago and you're thinking about adjusting their diuretic, well then you've got some useful information, (but not if) you have a potassium or creatinine that's three months old.

Another issue that arose during the trial was some of the automated alerts advised the patient to phone the Heart Function Clinic. However, someone was not always available to answer the phone, and no one answered the phone outside of regular work hours.

#### Being Watched Long-term and System Dependency

A very small minority of patients expressed not wanting to use the telemonitoring system long-term, even though they thought there was a benefit to using it for a few months in order to learn more about self-care. They did not like “being watched” long-term, because they wanted “to enjoy life once in a while”.

Patient #35 …I feel like a prisoner. It doesn't take much to gain three pounds. I used to drink three or four beers. Next day, I know my weight is going to be higher. I don't want a phone call, you know. It's kind of pressure... it's pressuring me, you know.

Another potential negative effect of using the telemonitoring system is that some patients may feel dependent on it.

Patient #29 …(Taking the monitoring system away is like) another crutch that you're losing. You become conditioned to having it there as a backup, and there would be an initial sort of sense of loss.

## Discussion

Our study provided an in-depth investigation into the perceptions and experiences of heart failure patients and clinicians on the use of a mobile phone-based telemonitoring system and the mechanisms that helped improve self-care and clinical management. For example, self-care improved, because the patients felt more aware and knowledgeable regarding their heart failure condition, less anxiety, and more empowered and motivated to improve their condition. However, both the clinicians and patients had some concerns on using the telemonitoring system long-term, such as obtaining operational funding for the telemonitoring system and potentially increased clinical workload. This study also provided insight into system characteristics that are essential for the success of telemonitoring on heart failure outcomes. In particular, these important characteristics are (1) immediate self-care feedback, (2) immediate clinical feedback as necessary, (3) ease of use, and (4) perceived benefits of the telemonitoring system for continued adherence. 

### Immediate Self-Care Feedback

Appropriate heart failure self-care has been found to improve health outcomes and reduce healthcare costs [12-14]. Self-care has also been stressed as an important component of heart failure management in international clinical guidelines [15-17]. Our trial indicated the use of the telemonitoring system improved self-care as measured through the Self-Care of Heart Failure Index (SCHFI) [18]. In particular, a comparison of the post-trial data between the telemonitoring and control groups found a statistically significant difference in SCHFI maintenance scores (*P*= .03), indicating the telemonitoring group had greater self-care maintenance (ie, a higher SCHFI maintenance score). From the patient interviews, it was evident they were able to modify their own lifestyle behaviors from the automated immediate feedback of the telemonitoring system and the timely self-care feedback from their clinicians.

The lack of real-time, self-care feedback may be one of the primary reasons previous telemonitoring trials, such as the Telemonitoring to Improve Heart Failure Outcomes (Tele-HF) Trial, have failed to show positive health outcomes [3]. The Tele-HF trial intervention was an interactive voice-response system the patient called to record heart failure symptoms and weight data. The trial found no reduction in mortality or hospital admissions, but the system was lacking real-time patient feedback on self-care instructions and alerts.

### Immediate Clinical Feedback

From the clinicians’ perspective, the telemonitoring system alerted them to contact their patients, such as for continuous optimization of medications. From the patients’ perspective, the immediate clinical feedback not only improved health outcomes, but also reduced their anxiety by knowing that someone was watching over them and motivated them to improve self-care. In the Tele-HF trial, site coordinators reviewed patient information daily during weekdays and should have contacted the patient as required. It is thus possible the response by clinicians to indications of decompensation was not timely.

### Ease of Use

Both patients and clinicians found the telemonitoring system to be easy to use. The system was highly automated and required minimal understanding of technology, including mobile phones. Previous trials of heart failure telemonitoring have demonstrated that when patients find the system difficult to use, adherence to taking the physiological measurements is poor. The MOBIle TELemonitoring in Heart Failure Study (MOBITEL) asked the intervention group (n = 54) to send their daily measurements of blood pressure, heart rate, body weight, and dosage of heart failure medication via a mobile phone’s Internet browser [19]. Due to the patients having difficulty in using the browser to transmit the data, even after intensive training, 12 patients dropped out immediately.

### Perceived Benefits

Participants in the current trial completed their daily measurements on average 5 to 6 days per week throughout the six-month duration. The high adherence to daily measurements can be partially attributed to the ease of use of the system. However, patients must also believe that transmitting daily information will benefit them in tangible ways. During the pre-trial patient interviews, many of the patients stated they stopped taking their blood pressure at home, because the readings were not sent to their clinicians and they did not know how to interpret or act on the blood pressure readings on their own [20].

The clinicians supported the use of telemonitoring, because they thought it was a useful tool to complement the management of their patients’ heart failure conditions and to help increase self-care. They believed telemonitoring would ultimately improve their patients’ health outcomes, including reducing the number of hospital admissions. Although there were concerns of implementing the telemonitoring system, such as increased workload, the clinicians were willing to try to resolve any barriers, because of the numerous perceived benefits from mobile phone-based telemonitoring.

### Previous Studies on Perceptions of Telemonitoring

Our study supported some findings from previous studies on the thoughts of patients and clinicians on telemonitoring for various chronic illnesses. For example, previous studies have found that ease of use, perceived tangible benefits, and cost-effectiveness are important aspects of mobile phone-based telemonitoring adoption for heart failure, asthma, and blood pressure management [20-23]. However, whether telemonitoring increases or decreases patient anxiety appears to be dependent on the particular patient population. A study on nocturnal home hemodialysis found anxiety reduced with telemonitoring, especially for the patient’s caregiver [24], which is similar to the findings from the present trial. Conversely, a study on blood pressure telemonitoring found that a major concern was increased patient anxiety, such as from a single high blood pressure reading [23]. Possibly, as the perception of the severity of the chronic illness or event being monitored increases, the more beneficial telemonitoring is at reducing anxiety.

### Limitations

A limitation to the findings from this study is their general applicability, because of the specific group of heart failure patients chosen to participate. The participants were all recruited from the Heart Function Clinic and, therefore, may have been more motivated to adhere to the telemonitoring protocol than patients followed by primary physicians. The participants were also younger on average than the average heart failure patient, because the UHN Heart Function Clinic treats a high proportion of severely ill patients, including some very young patients (ie, in their twenties). However, many of the study participants were elderly. In addition, many of the participants had stable heart failure (ie, had not been admitted into hospital for several years). Higher-risk patients, who are frequently readmitted to hospital, might benefit even more from telemonitoring. Further investigation into the characteristics of heart failure patients, who would be suitable for and benefit from mobile phone-based telemonitoring, is required [4].

Finally, clinicians who participated in the study may have been more willing to use the telemonitoring system than primary physicians and those at other heart function clinics. Prior to the trial, some of the clinicians used their own personal time to communicate with their patients and kept in close contact with their high-risk patients through various means (ie, telephone, email, and fax). Therefore, despite their concerns about potentially increased workload, they may have been more amenable to the idea of mobile phone-based telemonitoring.

### Conclusions

Mobile phone-based telemonitoring enabled patients to positively change their lifestyle behaviors and to improve their quality of life, including reducing anxiety. The telemonitoring system also improved clinical management, by providing real-time physiological information and alerts. The in-depth qualitative analysis revealed several system characteristics that contributed to improved heart failure management, such as (1) having immediate self-care and clinical feedback, (2) being easy and quick to use, and (3) providing tangible benefits to the end-users (ie, the patients). These system characteristics will help ensure patient adherence and clinician buy-in, which are both necessary for successful implementation of any telemonitoring solution.
